# A validated survival score for patients with metastatic spinal cord compression from non-small cell lung cancer

**DOI:** 10.1186/1471-2407-12-302

**Published:** 2012-07-20

**Authors:** Dirk Rades, Sarah Douglas, Theo Veninga, Steven E Schild

**Affiliations:** 1 Department of Radiation Oncology, University of Lubeck, Lubeck, Germany; 2 Department of Radiation Oncology, Dr. Bernard Verbeeten Institute, Tilburg, Netherlands; 3 Department of Radiation Oncology, Mayo Clinic Scottsdale, Scottsdale, AZ, USA; 4 Department of Radiation Oncology, University of Lübeck, Ratzeburger Allee 160, d-23538, Lübeck, Germany

**Keywords:** Non-small cell lung cancer, Metastatic spinal cord compression, Radiotherapy, Survival prognosis, Scoring system

## Abstract

**Background:**

This multicenter study aimed to create and validate a scoring system for survival of patients with metastatic spinal cord compression (MSCC) from non-small cell lung cancer (NSCLC).

**Methods:**

The entire cohort of 356 patients was divided in a test group (N = 178) and a validation group (N = 178). In the test group, nine pre-treatment factors including age, gender, Eastern Cooperative Oncology Group performance status (ECOG-PS), number of involved vertebrae, pre-radiotherapy ambulatory status, other bone metastases, visceral metastases, interval from cancer diagnosis to radiotherapy of MSCC, and the time developing motor were retrospectively analyzed.

**Results:**

On multivariate analysis, survival was significantly associated with ECOG-PS, pre-radiotherapy ambulatory status, visceral metastases, and the time developing motor deficits. These factors were included in the scoring system; the score for each factor was determined by dividing the 6-month survival rate (in %) by 10. The risk score represented the sum of the scores for each factor. According to the risk scores, which ranged from 6 to 19 points, three prognostic groups were designed. The 6-month survival rates were 6% for 6–10 points, 29% for 11–15 points, and 78% for 16–19 points (p < 0.001). In the validation group, the 6-month survival rates were 4%, 24%, and 76%, respectively (p < 0.001).

**Conclusions:**

Since the survival rates of the validation group were similar to those of the test group, this score can be considered reproducible. The scoring system can help when selecting the individual treatment for patients with MSCC from NSCLC. A prospective confirmatory study is warranted.

## Background

Non-small lung cancer (NSCLC) accounts for about 15% of all primary tumor types leading to metastatic spinal cord compression (MSCC) [1]. Treatment options for this oncologic emergency include different types and programs of radiotherapy alone or, for selected patients, upfront decompressive surgery followed by radiotherapy [2]. Selection of the optimal treatment approach for the individual patient should take into account the patient’s estimated survival time. Patients with a very poor survival prognosis are generally not candidates for a burdensome treatment including decompressive surgery or longer-course radiotherapy of two to four weeks. They appear better treated with single-fraction radiotherapy which means less discomfort for these debilitated patients. In contrast, patients with a more favorable survival prognosis may benefit from decompressive surgery or from longer-course radiotherapy programs, which lead to better local control of MSCC than single-fraction or short-course irradiation [3,4].

For optimal personalization of the treatment for each patient with MSCC, it is critical to regard patients with MSCC from a particular primary tumor type as a separate group of patients, because primary tumors vary with respect to their biological behavior. Therefore, the present study aimed to create and validate a survival score particularly for patients with MSCC from NSCLC, one of the most common primary tumor types in patients presenting with this oncologic complication.

## Results

The median survival time in the entire cohort was 4 months. The patients whose data were included in this study had been treated between 1992 and 2010. The 6-months survival rates were 25% for patients treated until 2005 (n = 262) and 34% for patients treated after 2005 (n = 94) (p = 0.39).

**Figure 1:**
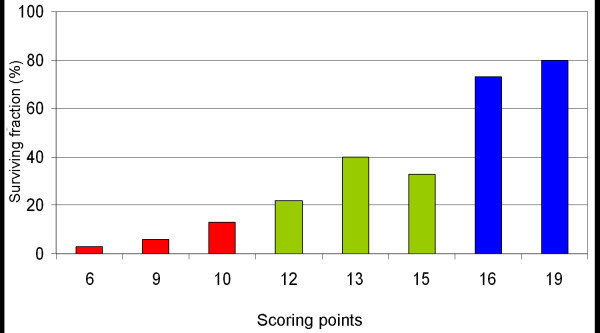
Test group: The total scores in relation to the 6-months survival rate (in %).

**Figure 2:**
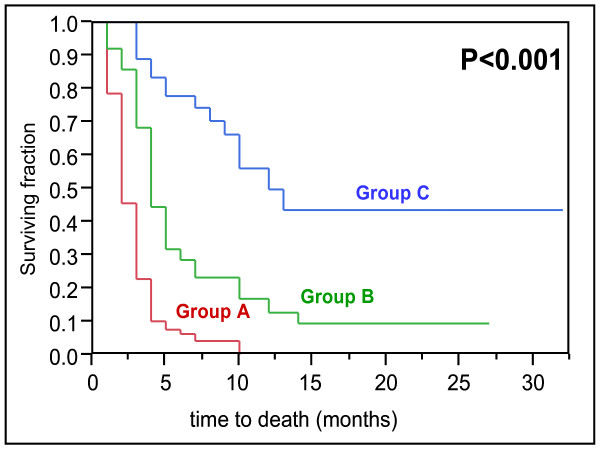
Kaplan-Meier curves for survival of the three score groups A (6–10 points), B (11–15 points), and C (16–19 points) from the test group.

**Figure 3:**
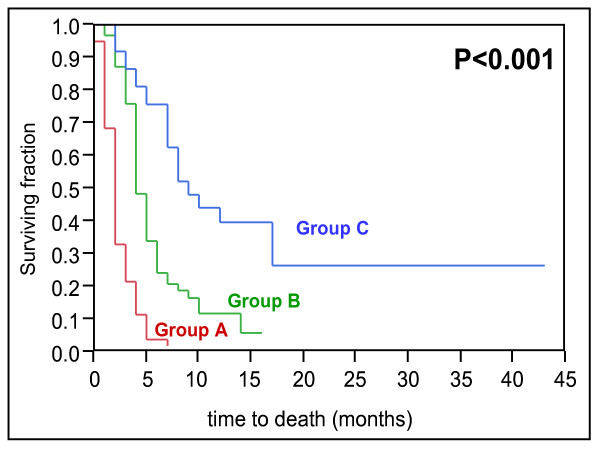
Kaplan-Meier curves for survival of the three score groups A (6–10 points), B (11–15 points), and C (16–19 points) from the validation group.

In the univariate analysis of the test group, survival was associated with the Eastern Cooperative Oncology Group performance status (ECOG-PS), number of involved vertebrae, pre-radiotherapy ambulatory status, other bone metastases, visceral metastases, and the time developing motor deficits. The results of this univariate analysis are given in Table 1. In the multivariate analysis of the test group, ECOG-PS, pre-radiotherapy ambulatory status, visceral metastases, and the time developing motor deficits maintained significance and were included in our scoring system. The results of the multivariate analysis are shown in Table 2. The scores for each of the four significant prognostic factors obtained from the 6-months survival rate are given in Table 3. The addition of the four scores for each factor resulted in total scores of 6, 9, 10, 12, 13, 15, 16, or 19 points (Figure 1). According to the total scores, the patients of the test group were divided into three risk groups: 6–10 points (group A, n = 79), 11–15 points (group B, n = 63), and 16–19 points (group C, n = 36). The 6-month survival rates were 6% for group A, 29% for group B, and 78% for group C (p < 0.001, Figure 2). The 6-months survival rates of the three risk groups of the validation group were 4%, 24%, and 76%, respectively (p < 0.001, Figure 3).

**Table 1:** Test group: Univariate analysis of pre-treatment factors and the radiation regimen for survival

	**Survival**	**Survival**	**Median**	**p-value**
	**at 6 months (%)**	**at 12 months (%)**	**survival time (months)**	
**Age**				
≤ 64 years	28	15	3	
≥ 65 years	30	15	4	0.50
**Gender**				
Female	32	29	4	
Male	27	10	4	0.13
**ECOG Performance status**				
1-2	51	26	6	
3-4	15	7	3	**<0.001**
**Number of involved vertebrae**				
1-2	44	28	4	
≥ 3	19	4	3	**<0.001**
**Ambulatory status prior to RT**				
Not Ambulatory	12	7	2	
Ambulatory before RT	44	21	5	**<0.001**
**Other bone metastases**				
No	45	30	4	
Yes	19	4	3	**<0.001**
**Visceral metastases**				
No	56	38	9	
Yes	15	3	3	**<0.001**
**Interval from cancer diagnosis to radiotherapy of MSCC**				
≤ 15 months	26	13	3	
> 15 months	40	23	5	0.16
**Time developing motor deficits**				
1-7 days	12	4	2	
> 7 days	40	22	5	**<0.001**
**Radiation regimen**				
Short-course radiotherapy	24	10	3	
Longer-course radiotherapy	32	18	4	0.12

**Table 2:** Test group: Multivariate analysis of pre-treatment factors and the radiation regimen for survival

	**Risk ratio**	**95%-confidence interval**	**p-value**
**ECOG Performance status**	1.98	1.36 – 2.91	**<0.001**
**Number of involved vertebrae**	1.17	0.89 – 1.56	0.27
**Ambulatory status before radiotherapy**	1.65	1.05 – 2.63	**0.029**
**Other bone metastases**	1.16	0.63 – 2.10	0.63
**Visceral metastases**	2.44	1.66 – 3.68	**<0.001**
**Time developing motor deficits**	1.33	1.12 – 1.57	**0.001**

**Table 3:** Test group: 6-month survival rates and corresponding scores

	**Survival**	**Score**
	**at 6 months (%)**	**(points)**
**ECOG Performance status**		
1-2	51	5
3-4	15	2
**Ambulatory status before radiotherapy**		
Not Ambulatory	12	1
Ambulatory before RT	44	4
**Visceral metastases**		
No	56	6
Yes	15	2
**Time developing motor deficits**		
1-7 days	12	1
> 7 days	40	4

## Discussion

Personalization of cancer treatment, which has become more important in oncology during recent years, must take into account the patient’s life expectancy. This accounts in particular for a palliative situation such as MSCC. Survival scores help estimate the survival prognosis of each patient. Several scores created to estimate the survival of patients with bone metastases already exist. A few prognostic scores have been developed in particular for patients with bone metastases in the vertebral column.

The majority of these scores were designed by to help surgeons decide whether spinal surgery may be indicated or not. In 1990, Tokuhashi et al. presented a score based on the data of 64 patients with a metastatic spine tumor who underwent spinal surgery [5]. Their score has been revised 15 years later in a series of 246 patients [6]. Bauer et al. reported a scoring system including scoring for pathological fracture based on the data of 88 patients with spinal metastasis plus 153 patients with bone metastasis of the extremities in 1995 [7]. Leithner et al., who compared different scoring systems in their series of 69 patients in 2008, suggested a modified Bauer score without scoring for pathological fracture [8]. The Tomita score presented in 2001 included the data of 67 patients [9]. Except the revised Tokuhashi score [6], these scoring systems may have a limited validity due to the relatively small number of patients included. Furthermore, these scores were designed for patients with spinal metastasis in general, and not particularly for patients with motor deficits due to MSCC.

In 2005, Van der Linden et al. presented a score in a larger series of patients (n = 342) with painful spinal metastases who had received radiotherapy alone and no surgery [10]. In that study, patients with neurologic impairment were not included. All these previous scoring systems included patients with spinal metastasis from many different primary tumors. However, because various primary tumor types behave differently, it is important to have separate scores for the different tumor entities, in particular for the most common ones such as breast cancer, prostate cancer, and NSCLC [1].

In the present study, four independent prognostic factors were found to be significantly associated with survival in patients with MSCC from NSCLC in a comparably large series of patients. These significant factors included the ECOG-PS, pre-radiotherapy ambulatory status, visceral metastases, and the time developing motor deficits. In our previous report on prognostic factors for different outcomes in the entire cohort of 356 patients, gender, other bone metastases, and the interval from the first diagnosis of NSCLC to radiotherapy of MSCC were also significantly associated with survival [11]. However, we included only those prognostic factors found to be independent in the multivariate analysis of the test group in the present score, because we felt that this would make the score more robust.

When compared to MSCC from other solid tumors, patients with MSCC from NSCLC have a less favorable estimated survival [1]. This is reflected by the fact that the worst prognostic group, group A, was the largest group in the present study. Based on the 6-months survival times related to the four independent prognostic factors, three prognostic groups were formed. Group A patients had the worst prognosis, only 6% of patients in the test group and 4% in the validation group survived at least 6 months following irradiation. These patients may be considered candidates for single-fraction radiotherapy or even best supportive care alone. Group B patients had 6-months survival rates of 29% and 24%, respectively and may be treated with short-course multi-fraction radiotherapy such as 20 Gy in 5 fractions over one week. Short-course radiotherapy is as effective as longer programs with respect to post-radiotherapy motor function [12]. In contrast, local control of MSCC is better with longer-course than with short-course radiotherapy [3,4]. However, local control of MSCC appears of minor importance in group B patients, because most of these patients will not live long enough to experience a local recurrence of MSCC. In contrast, group C patients who achieved 6-months survival rates of 78% and 76%, respectively, are at a higher risk of developing a local recurrence of MSCC and, therefore, are likely to benefit from longer-course radiotherapy such as 10x3 Gy in 2 weeks or 20x2 Gy in 4 weeks. In group B and group C patients, upfront decompressive surgery in addition to radiotherapy may be reasonable for selected patients with a good performance status and involvement of only one spinal segment. This accounts in particular for patients who are unlikely to be able to walk after radiotherapy alone. In a randomized trial of 101 patients, decompressive surgery followed by radiotherapy led to better pre-radiotherapy ambulatory function and survival than radiotherapy alone in such patients [2].

The present score focused on a single tumor entity. In contrast, previous prognostic indices for patients with vertebral metastases or other palliative situations included many different tumor types [5-10,13-17]. Therefore, the present scoring system takes more into account the patient’s individual situation. In order to validate our score, the risk groups A, B and C of the test group were compared to the corresponding groups A, B and C of the validation group. The 6-months survival rates of the three groups in the validation group proved to be similar to the corresponding 6-months survival rates in the test group. Thus, this new score for MSCC from NSCLC appears valid and reproducible. However, the score is based on retrospective data. Furthermore, data on systemic treatment following treatment was not available in most patients. These two aspects may have led to a hidden selection bias. Therefore, the results of the present study need to be confirmed in a prospective series of patients.

## Conclusions

The present survival score for patients with MSCC from NSCLC was based on four independent prognostic factors and included three prognostic groups. Patients of group A have the worst prognosis and may be candidates for single-fraction radiotherapy or even best supportive care alone. Patients of group B may be treated with short-course multi-fraction radiotherapy, and patients of group C, who have the most favorable prognosis, appear best treated with longer-course radiotherapy. For selected patients of groups B and C, upfront decompressive surgery in addition to radiotherapy may be considered. The decision for or against decompressive surgery requires an additional scoring system taking into accout the functional outcome following radiotherapy alone. Regarding the score presented here, a prospective confirmatory study is warranted.

## Methods

Three-hundred-and-fifty-six unselected patients with treated with MSCC from NSCLC were retrospectively analyzed in this multicenter study. The patients had received radiotherapy alone for MSCC-related motor deficits of the legs. Patients who had prior surgery or radiotherapy to the currently involved parts of the spinal cord were not included. The majority of the patients (n = 262) were treated unitl 2005, i.e. before the randomized study of Patchell et al. comparing radiotherapy alone to decompressice surgery followed by radiotherapy was published [2]. Of the 94 patients treated between 2006 and 2010, only 16 patients (4% of the entire cohort) would have met the inclusion criteria of the Patchell study. Thus, the risk of a selection bias due to excluding patients receiving surgery appears relatively low.

Adequate diagnostic imaging including spinal CT or spinal MRI was requested, as well as corticosteroid treatment during radiotherapy. Patients were presented to a surgeon prior to radiotherapy to discuss the option of upfront decompressive surgery when indicated. The data were collected from the patients, their treating physicians, and the patient files. Because this study did not report on a clinical trial, and because the data were retrospective in nature and analyzed anonymously, approval by an ethic committee was not necessary. Radiotherapy was performed with 6–10 MeV photon beams from a linear accelerator. The treatment volumes generally encompassed one normal vertebra above and below the involved vertebrae. One-hundred-and-forty-three patients had received short-course radiotherapy (1x8 Gy or 5x4 Gy in 1 week), and 213 patients were treated with longer-course radiotherapy (10x3 Gy in 2 weeks, 14-15x2.5 Gy in 3 weeks, or 20x2 Gy in 4 weeks).

The 356 patients were alternately assigned to the test group (N = 178) or the validation group (N = 178). The characteristics of both patient groups are given in Table 4. In the test group, nine pre-treatment factors were investigated including age (≤64 vs. ≥65 years; median age: 64 years), gender, ECOG-PS (1–2 vs. 3–4), number of involved vertebrae (1–2 vs. ≥3), pre-radiotherapy ambulatory status (not ambulatory vs. ambulatory), other bone metastases prior to radiotherapy (no vs. yes), visceral metastases prior to radiotherapy (no vs. yes), interval between first diagnosis of NSCLC and radiotherapy of MSCC (≤15 vs. >15 months, in accordance with previous studies), and time of developing motor deficits prior to radiotherapy (1–7 vs. >7 days, in accordance with previous studies). Because 69 patients of the 194 patients (36%) who were ambulatory prior to radiotherapy in the entire cohort had a poor ECOG-PS of 3–4, both performance status and pre-radiotherapy ambulatory status were investigated. In addition to these pre-treatment factors, the potential impact of the radiation regimen (short-course vs. longer-course radiotherapy) has been investigated. The univariate analysis of survival was performed with the Kaplan-Meier-method and the log-rank test [18]. The significant prognostic factors (p < 0.05) were additionally evaluated in a multivariate analysis performed with the Cox proportion hazards model. The prognostic factors that were significant in the multivariate analysis of the test group were included in the scoring system. The score for each significant prognostic factor was determined by dividing the 6-month survival rate (in %) by 10. The total score represented the sum of the scores for each factor.

**Table 4:** Patient characteristics of the test group and the validation group. The p-values were obtained from the Chi-square test

	**Test group**	**Validation group**	**p-value**
	**n patients (%)**	**n patients (%)**	
**Age**			
≤ 64 years	97 (54)	94 (53)	
≥ 65 years	81 (46)	84 (47)	0.89
**Gender**			
Female	47 (26)	45 (25)	
Male	131 (74)	133 (75)	0.92
**ECOG Performance status**			
1-2	67 (38)	66 (37)	
3-4	111 (62)	112 (63)	0.96
**Number of involved vertebrae**			
1-2	71 (40)	77 (43)	
≥ 3	107 (60)	101 (57)	0.73
**Ambulatory status prior to RT**			
Not Ambulatory	84 (47)	78 (44)	
Ambulatory before RT	94 (53)	100 (56)	0.73
**Other bone metastases**			
No	67 (38)	76 (43)	
Yes	111 (62)	102 (57)	0.58
**Visceral metastases**			
No	61 (34)	56 (31)	
Yes	117 (66)	122 (69)	0.81
**Interval from cancer diagnosis to radiotherapy of MSCC**			
≤ 15 months	148 (83)	150 (84)	
> 15 months	30 (17)	28 (16)	0.92
**Time developing motor deficits**			
1-7 days	74 (42)	73 (41)	
> 7 days	104 (58)	105 (59)	0.95
**Radiation regimen**			
Short-course radiotherapy	71 (40)	72 (40)	
Longer-course radiotherapy	107 (60)	106 (60)	0.97

## Abbreviations

ECOG, Eastern Cooperative Oncology Group; ECOG-PS, Eastern Cooperative Oncology Group performance score; Gy, Gray; MeV, Mega electron volts; MSCC, Metastatic spinal cord compression; RT, Radiotherapy.

## Competing interests

The authors declare that they have no competing interests.

## Authors’ contributions

DR and SES participated in the design of the study. SES performed the statistical analyses. SD, TV, and DR provided study materials. All authors were involved in manuscript writing; they read and approved the final manuscript.

## Pre-publication history

The pre-publication history for this paper can be accessed here:

http://www.biomedcentral.com/1471-2407/12/302/prepub
